# Supplementary material for: Dietary Supplemental Chromium Yeast Improved the Antioxidant Capacity, Immunity and Liver Health in Broilers under High Stocking Density
Source: Animals (Basel). 2022 Aug 28;12(17):2216. doi: 10.3390/ani12172216 (PMC9454686; doi:10.3390/ani12172216)
Supplement: Supplementary file 1 [file animals-12-02216-s001.zip › animals-1855442-supplementary.pdf]

Table S1 The dietary nutrients and Cr contents of different treatment groups

Items[1]	LSD	HSD	HSD + Cr-yeast (μg Cr/kg)			
			200	400	800	1600
CP (%)	19.05	19.05	19.00	19.09	19.05	19.13
Ca (%)	0.93	0.93	0.93	0.89	0.90	0.87
P (%)	0.41	0.41	0.41	0.49	0.48	0.49
Lysine (%)	1.12	1.12	1.14	1.09	1.14	1.14
Methionine (%)	0.53	0.53	0.56	0.54	0.54	0.57
Threonine (%)	0.71	0.71	0.73	0.74	0.71	0.74
Tryptophan (%)	0.19	0.19	0.23	0.19	0.22	0.21
Cysteine (%)	0.27	0.27	0.26	0.24	0.25	0.23
Cr (μg/kg)	468.47	468.47	668.47	834.99	1204.09	2324.63

[1] LSD, low stocking density; HSD, high stocking density; CP, crude protein; Ca, calcium; P, phosphorus

Table S2. The optimal addition levels of Cr-yeast in broilers under high stocking density.

Items		Broken-line equation	R^2	Optimal addition level (μg Cr/kg)
Liver health	ALT (ng/L)	y=93.18+0.021(1531.6-x)	0.9525	1531.60
	ALP (ng/L)	y=166.6+0.0135(800-x)	0.9976	800.00
Serum immunity	IL-10 (ng/L)	y=40.304+0.0313(319.3-x)	0.9999	319.30
	T3 (pmol/L)	y=234.7+0.0211(961-x)	0.9742	961.00
Antioxidant Status	Serum			
	SOD (pg/mL)	y=34.4083+0.0039(490-x)	0.9861	490.00
	GSH-Px (pmol/mL)	y=13.952-0.00287(665-x)	0.9993	665.00
	Liver			
	MDA (nmol/mgprot)	y=1.316+0.00475(579-x)	0.9626	579.00
	Breast			
	T-AOC (U/mgprot)	y=0.136-0.00045(425-x)	0.9868	425.00
	CAT (U/mgprot)	y=0.718-0.00004(466.7-x)	0.9833	466.70

R^2 represents the fitting degree of broken-line regression. The closer R2 is to 1, the better the fitting degree. Therefore, we selected the indicators closer to 1 ($R^2 > 0.95$) to obtain the break point. Some indicated of $R^2 < 0.95$ had been deleted in Table S1. The optimal supplementation of Cr-yeast in liver health, immunity ability and antioxidant capacity were at the range of 800.00 - 1531.60, 319.30 - 961.00, and 425.00 - 665.00 μg Cr/kg respectively.

Table S3. Effects of Cr-yeast on antioxidant status of broilers under high stocking density [1].

Item[2]	LSD	HSD	HSD + Cr-yeast (μg Cr/kg)				SEM	*p*-Value[3]		
			200	400	800	1600		ANOVA	Linear	Quadratic
TNF-α (ng/L)	31.25[abc]	31.44[ab]	27.96[bc]	33.83[a]	26.78[c]	27.97[bc]	0.694	0.010	0.017	0.008
IL-1β (ng/L)	113.09[d]	113.74[d]	128.31[a]	117.44[c]	109.52[e]	124.75[b]	1.153	<0.001	<0.001	<0.001
IL-10 (ng/L)	42.20[b]	42.98[b]	36.72[d]	39.34[c]	44.37[a]	38.11[c]	0.495	<0.001	<0.001	<0.001
T3 (pmol/L)	287.65[b]	240.10[d*]	263.88[c]	269.99[c]	303.01[a]	209.22[e]	5.263	<0.001	<0.001	<0.001
T4 (pmol/L)	1078.59[c]	1184.21[a*]	871.06[e]	998.92[d]	1073.65[c]	1149.00[b]	17.754	<0.001	<0.001	<0.001

[1] Values are expressed as means of six replicates per treatment.

[2] LSD, low stocking density; HSD, high stocking density; TNF-α, Tumor necrosis factor-α; IL-1β, Interleukin-1β; IL-10, Interleukin-10; T3, thyroxine; T4, thyrotropine; SEM, standard error of means.

[3] Results of linear and quadratic analysis of five high stocking density groups.

[*] Means t-test results of independent samples in group LSD and group HSD ($p^* < 0.05$, $p^{**} < 0.01$).

[a-d] Means in the same row without the same superscripts differ significantly ($p < 0.05$).
